# Fungal naphtho-*γ*-pyrones: Potent antibiotics for drug-resistant microbial pathogens

**DOI:** 10.1038/srep24291

**Published:** 2016-04-11

**Authors:** Yan He, Jun Tian, Xintao Chen, Weiguang Sun, Hucheng Zhu, Qin Li, Liang Lei, Guangmin Yao, Yongbo Xue, Jianping Wang, Hua Li, Yonghui Zhang

**Affiliations:** 1 Hubei Key Laboratory of Natural Medicinal Chemistry and Resource Evaluation, School of Pharmacy, Tongji Medical College, Huazhong University of Science and Technology, Wuhan 430030, China; 2 Department of Physical Medicine and Rehabilitation, Zhongnan Hospital of Wuhan University, Wuhan 430071, China

## Abstract

Four naphtho-*γ*-pyrones (fonsecinones A and C and aurasperones A and E) were identified as potential antibacterial agents against *Escherichia coli*, extended-spectrum *β*-lactamase (ESBL)-producing *E. coli, Pseudomonas aeruginosa, Enterococcus faecalis*, and methicillin-resistant *Staphylococcus aureus* (MRSA) in an *in vitro* antibacterial screen of 218 fungal metabolites. Fonsecinone A (**2**) exhibited the most potent antibacterial activity, with minimum inhibitory concentrations (MICs) of 4.26, 17.04, and 4.26 *μ*g/mL against ESBL-producing *E. coli, P. aeruginosa*, and *E. faecalis*, respectively. The inhibitory effects of fonsecinones A (**2**) and C (**3**) against *E. coli* and ESBL-producing *E. coli* were comparable to those of amikacin. Molecular docking-based target identification of naphtho-*γ*-pyrones **1**–**8** revealed bacterial enoyl-acyl carrier protein reductase (FabI) as an antibacterial target, which was further validated by FabI affinity and inhibition assays. Fonsecinones A (**2**) and C (**3**) and aurasperones A (**6**) and E (**7**) bound FabI specifically and produced concentration-dependent inhibition effects. This work is the first report of anti-drug-resistant bacterial activities of naphtho-*γ*-pyrones **1**–**8** and their possible antibacterial mechanism of action and provides an example of the successful application of *in silico* methods for drug target identification and validation and the identification of new lead antibiotic compounds against drug-resistant pathogens.

The emergence and spread of antibiotic-resistant bacteria have potentially drastic consequences for human health globally^1^. Methicillin-resistant *Staphylococcus aureus* (MRSA), *Enterococcus faecalis* (*E. faecalis*), extended-spectrum β-lactamase-producing *Escherichia coli* (ESBL-producing *E. coli*), *Klebsiella pneumoniae*, and *Pseudomonas aeruginosa* express a remarkable array of resistance and virulence factors, which contributes to their prominent roles in hospital-acquired infections^2^. New antibiotics or for further strategies to overcome antibiotic resistance are thus urgently needed. Microorganisms produce diverse antibiotics that function in an antagonistic capacity in nature in competition with other organisms^3^, and most antibacterial agents in clinical or preclinical trials are either microbial products or analogs^3^,^4^,^5^. Another effective approach is drug repurposing, in which new useful activities of old drugs are identified by screening against relevant disease targets^6^. *Aspergillomarasmine* A, a fungal metabolite discovered 50 years ago, was recently repurposed based on its ability to overcome antibiotic resistance associated with metallo-*β*-lactamase^7^. The development of bioinformatic and computational biology techniques will greatly facilitate, this type of drug repurposing^8^,^9^,^10^.

Our efforts to identify novel antibiotics from microbial sources yielded four naphtho-*γ*-pyrones (compounds **2**, **3**, **6**, and **7**) that possess potent inhibitory activities against MRSA and ESBL-producing *E. coli in vitro*. We then analyzed the antimicrobial activities and possible mechanisms of action of relevant analogues (compounds **1**–**8**) isolated by our group in detail. Naphtho-*γ*-pyrones are an important group of fungal polyketides, some of them have been shown to exert antimicrobial activities against *S. aureus, B. cereus, M. tuberculosis, E. coli*, plant pathogenic bacteria, and *C. albicans*^11^,^12^,^13^,^14^. However, few studies have focused on the activities of naphtho-*γ*-pyrones against drug-resistant bacteria^2^, their mechanisms of action and structure-activity relationships^14^,^15^.

In this study, the antimicrobial effects of naphtho-*γ*-pyrones **1**–**8** against MRSA, ESBL-producing *E. coli, K. pneumoniae, P. aeruginosa*, and *E. faecalis* were investigated. We also performed structure-based virtual screening and confirmatory bioassays to identify the possible antibacterial targets of these bioactive naphtho-*γ*-pyrones.

## Results and Discussion

### The structure of the naphtho-*γ*-pyrones

The structures of compounds **1**–**8** were determined by ^1^H NMR, ^13^C NMR, MS, and ECD data analysis and by comparison with the literature^16^,^17^,^18^,^19^,^20^. Compounds of this type usually display axial chirality due to the high energy barrier for rotation of the bond linking the individual aromatic systems^19^,^21^. According to the literature^22^,^23^, (*R*)-configured dimeric naphtho-*γ*-pyrones exhibit a negative Cotton effect in the long-wavelength region and a positive one at shorter wavelengths. The ECD spectra of compounds **1**–**8** (see Fig. S20, Supporting Information) suggested that the absolute configurations of both compounds were (*R*). Moreover, the calculated ECD spectra of compounds **3**, **4**, and **7** matched well with their experimental ECD spectra (see Figs S21–S23, Supporting Information), confirming the structures and configurations of the investigated compounds.

### Antimicrobial activity

**Figure 1:**
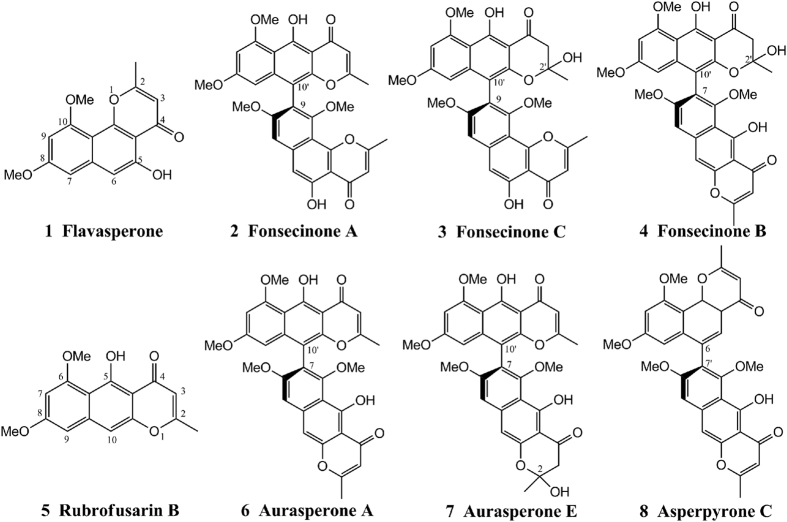
Chemical structures of the investigated naphtho-*γ*-pyrones **1**–**8**.

MRSA (ATCC 43300) and ESBL-producing *E. coli* (ATCC 35218) were used to screen antibacterial activity against antimicrobial-resistant pathogenic bacteria^2^. A total of 218 fungal metabolites isolated by our research group were screened *in vitro*. Fonsecinones A (**2**) and C (**3**) and aurasperones A (**6**) and E (**7**) exhibited potent inhibitory activities against MRSA and ESBL-producing *E. coli* at 100 *μ*g/mL. The polycyclic skeletons of these compounds (Fig. 1) are similar to that of naphtho-*γ*-pyrone. Compounds **2**, **3**, **6**, and **7** and four other compounds with similar structures—flavasperone (**1**), fonsecinone B (**4**), rubrofusarin B (**5**), and asperpyrone C (**8**)—were purified by HPLC (≥95%) for further study to confirm this result. Flavasperone (**1**) and rubrofusarin B (**5**) are monomers with linear and angular polycyclic skeletons^14^, respectively. Based on their diaryl bond connections, the six dimeric naphtho-*γ*-pyrones were classified as asperpyrone-type naphtho-*γ*-pyrones with C-10′-C-9 (**2** and **3**), C-10-C-7′ (**6**, **7**, and **8**), or C-6-C-7′ (**4**) linkages^13^,^14^ (Fig. 1). Further antibacterial assays using the serial dilution method were performed on MRSA (ATCC 43300), ESBL-producing *E. coli* (ATCC 35218), and four common nosocomial infection strains: *P. aeruginosa* (ATCC 27853), *K. pneumoniae* (ATCC 700603), *E. faecalis* (ATCC 29212), and *E. coli* (ATCC 29212). The MICs of the tested compounds against these bacterial are presented in Table 1. The activities of reference compounds recommended by the National Committee for Clinical Laboratory Standards (NCCLS) were also included for comparison^24^.

Most of the investigated naphtho-*γ*-pyrones displayed significant antibacterial activity against gram-negative bacteria. Fonsecinones A (**2**) and C (**3**) and aurasperones A (**6**) and E (**7**) possessed potential antibacterial activities against *E. coli*, ESBL-producing *E. coli, P. aeruginosa, E. faecalis* and MRSA in the micromolar range. Fonsecinone A (**2**) exhibited the most potent antibacterial activity. The MICs of Fonsecinone A for ESBL-producing *E. coli, P. aeruginosa* and *E. faecalis* were 4.26, 17.04, and 4.26 *u*g/mL, respectively. The inhibitory effects of fonsecinones A (**2**) and C (**3**) against *E. coli* and ESBL-producing *E. coli* were comparable to those of amikacin^24^, which exhibits MICs of 2.13 and 4.26 *μ*g/mL, respectively. The structure-activity relationships in these compounds indicated that the compounds with C-10′-C-9 linkages, fonsecinones A and C, had the highest antibacterial activities against the targeted pathogenic bacteria, followed by the compounds with C-10′-C-7 linkages, fonsecinone B and aurasperones A and E. Asperpyrone C, which features a C-6-C-7′ linkage, had the lowest antibacterial activities. The removal of electron-donating substituents (OH) at C2/C2′ was associated with higher antibacterial activity for these naphtho-*γ*-pyrones. Flavasperone, a monomer with an angular skeleton, exhibited higher antibacterial activity than its linear isomer (**5**).

### Identification of FabI as a possible antibacterial target by inverse docking

Compounds **1**–**8**, which exhibited potent antimicrobial activity, were subjected to further investigation to determine their possible antimicrobial mechanisms. A thorough literature survey indicated that several flavones^25^ and flavonoids^26^,^27^ with similar polycyclic skeleton structures and cephalochromin^28^, a bis-naphtho-*γ*-pyrone with a chaetochromin-skeleton, are inhibitors of cellular fatty acid biosynthesis in bacteria. Bacterial fatty acid biosynthesis enzymes are excellent targets for novel antibacterial agent discovery because of their essential roles in the synthesis of the cell membrane^29^. Several antibiotics, such as diazaborines, isoniazid, and triclosan, inhibit fatty acid synthetic enzymes^29^. Selective targeting of key bacterial fatty acid metabolism enzymes was performed via *in silico* target identification by small-scale inverse docking of 14 enzymes involved in fatty acid metabolism^29^,^30^ (Fig. S25) with compounds **1**–**8**. Targets with lower calculated binding energies are considered to have higher binding affinities for a specific compound^31^,^32^. The docking score results are presented in Table 2. FabI^29^, a single enoyl-ACP reductase enzyme that is the key control point within the bacterial fatty acid elongation cycle, was predicted to exhibit significantly higher binding affinities for the investigated naphtho-*γ*-pyrones. As the negative control, four unrelated targets^33^,^34^,^35^,^36^ were also performed docking with compounds **1**–**8**, the results were shown in Table S1, no significant binding was predicted for these targets.

**Figure 2:**
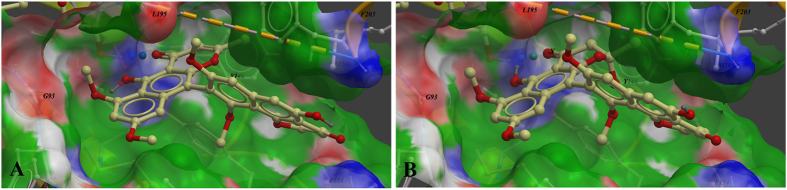
Low-energy binding conformations of (**A**) fonsecinone A and (**B**) fonsecinone C bound to *E.coli* FabI generated by virtual ligand docking. The structure of FabI was depicted in ribbon form. Fonsecinones A and C are depicted as the ball-and-stick model showing carbon (yellow), hydrogen (grey), oxygen (red) atoms.

FabI is highly conserved among most bacteria, including *E. coli, S. aureus, E. faecalis* and *P. aeruginosa*, and has no homologue in mammals^30^,^37^. FabI is therefore an attractive target for antibacterial drug development^29^,^37^. Notably, fonsecinones A and C, which possessed the most potent antibacterial activities, had the lowest binding energies to FabI (−37.40 and −35.27 kcal/mol, respectively). Representative complexes of the top scoring compounds are shown in Fig. 2. Similar to triclosan and other inhibitors observed in co-crystal structures^38^,^39^, fonsecinones A (Fig. 2A) and C (Fig. 2B) were well accommodated in the active site of FabI in the proposed binding mode^31^,^38^. The compounds formed key hydrophobic interactions with Gly93, Tyr146, Tyr156, Phe203, and Met206 of FabI. In addition, the compounds formed hydrogen bonds with the 2′-hydroxyl group of NAD^+^ and exhibited a key π-π accumulation effect on the nicotine ring of NAD^+ ^38,39.

### Bioactive naphtho-*γ*-pyrones maintain specific binding with FabI

**Figure 3:**
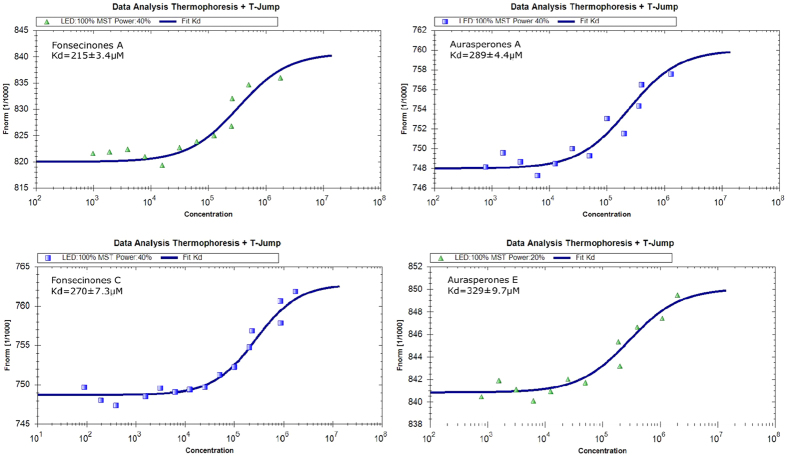
MST confirmed that compounds **2**, **3**, **6** and **7** maintained specific binding to *E. coli* FabI. Measurement of affinity between compounds **2**, **3**, **6** and **7** with FabI by MST in standard treated capillaries, the resulting binding curve was shown. From the resulting binding curve, Kd of 215 ± 3.4 *μ*M for fonsecinone A, 270 ± 7.3 *μ*M for fonsecinone B, 289 ± 31.1 *μ*M for aurasperone A, 329 ± 29.7 *μ*M for aurasperone E were calculated.

The ligand-protein networks suggested that the bioactive naphtho-*γ*-pyrones could bind FabI. Microscale thermophoresis (MST) was then performed to quantitatively measure binding^40^. The overall structures of FabI in *E. coli* and other pathogens have been reported to be very similar^29^; therefore, we expressed and purified *E. coli* FabI to quantify the dissociation constant (K_d_) for compound binding to the protein. Fonsecinones A (**2**) and C (**3**) and aurasperones A (**6**) and E (**7**) had K_d_ values of 215 ± 28.8, 270 ± 9.1, 289 ± 31.1, and 329 ± 29.7 *μ*M, respectively (Table 3), confirming their specific binding to *E. coli* FabI (Fig. 3).

### Bioactive naphtho-*γ*-pyrones inhibit FabI in a concentration-dependent manner

To further characterize their activity against FabI, compounds **1**–**8** were assayed. Six naphtho-*γ*-pyrones (**1**–**4, 6**, and **7**) inhibited *E. coli* FabI; their IC_50_ values are presented in Table 3. Fonsecinone A (**2**) exhibited the most potent inhibition, with an IC_50_ of only 2.78 *μ*M. The order of inhibitory potencies was **2** > **3** > **6** > **7** > **1** > (≈) **4**. The antibacterial activities of these compounds generally agreed with the experimentally determined FabI inhibitory activities. Among these compounds, fonsecinones A and C, had moderate antibacterial activities against *E. coli* and *S. aureus*, which express FabI as the sole enoyl reductase in the FASII system; however, no obvious inhibition of *K. pneumonia*, which produces only FabK, was observed^29^,^38^. The inhibitory activity of fonsecinone A against *E. coli* FabI was comparable to that of cephalochromin^28^, possibly indicating that the abilities of fonsecinone A and cephalochromin to access the active site of *E. coli* FabI and exert selective inhibitory effects are comparable, as observed in the molecular docking experiment (Figs 2 and S26). Fonsecinone A and cephalochromin have similar modes of binding in the active site of FabI and a potent π-π interaction with the nicotine ring of NAD^+ ^38,39. The results of the FabI inhibition assay are in agreement with the predicted binding free energies (Table 2), further validating the success of the inverse-docking target prediction and confirming FabI as an antibacterial target of the investigated naphtho-*γ*-pyrones.

In conclusion, a combination of *in vitro* antibacterial screening and molecular docking techniques were employed to identify several bioactive naphtho-*γ*-pyrones (**2**, **3**, **6**, and **7**) as potent antibiotics against a panel of drug-resistant microbial pathogens. These compounds exhibit FabI inhibition as one antibacterial mode of action. Notably, fonsecinones A and C (**2** and **3**) are both potent FabI inhibitors of fungal origin and exhibited promising antibacterial activities against the nosocomial infection pathogens MRSA, *P. aeruginosa, E. faecalis, E. coli*, and ESBL-producing *E. coli*, with MIC values in the micromolar range. These bioactive naphtho-*γ*-pyrones may be suitable as new antibiotics because of their potent antimicrobial properties and low toxicities^15^,^17^. The active naphtho-*γ*-pyrones found in this study may provide novel chemical scaffolds for the discovery of antibacterial agents.

## Materials and Methods

### General experimental procedures

UV spectra were measured on a Shimadzu UV-2401A spectrophotometer. IR spectra were determined on a Bruker Vertex 70 FT-IR spectrophotometer. ECD spectra were obtained with a JASCO J-810 spectrometer. HRESIMS was performed on an APIQSTAR Pulsar spectrometer mass spectrometer. NMR spectra were recorded on a Bruker AM–400 NMR spectrometer, and chemical shifts were referenced to the solvent peaks for CDCl_3_ (*δ*_H_ 7.26/*δ*_C_ 77.16). Semi-preparative HPLC was conducted on an Agilent 1100 liquid chromatography apparatus with a C_18_ column (5 *μ*m, 10 × 250 mm, YMC^TM^ Prep C_18_) and variable wavelength scanning ultraviolet detector (wavelength range: 190–600 nm). MPLC was conducted on a QuikSep-50 chromatography system (H&E Co., Ltd, Beijing, China). TLC was performed on silica gel GF254 (Qingdao Marine Chemical, Inc., Qingdao, China). Column chromatography were performed using silica gel (100–200 mesh and 200–300 mesh, Qingdao Marine Chemical Inc., Qingdao, China), ODS (50 *μ*m, YMC, Kyoto, Japan), and Sephadex LH–20 (Pharmacia Biotech AB, Uppsala, Sweden). The ELISA reader used SOFTmax PRO software (Molecular Devices, California, USA).

### Isolation of fungal natural products

Compounds **1**–**8** were isolated from *Aspergillus* sp. Z120. The strain was obtained from the Marine Culture Collection of China (MCCC, Xiamen, China) and was isolated from the Pacific Ocean. The DNA sequence data for the fungus have been deposited in GenBank under the accession number FJ798688. All information and strains collected can be obtained from the website http://www.mccc.org.cn/ and the collection center. The fungus was cultured in three 500-mL Erlenmeyer flasks each containing 100 mL of PDB at 20 °C on a rotary shaker (110 rpm) for 8 d to obtain the seed culture. Then, the seed culture was added to 500-ml Erlenmeyer flasks, each containing solid rice medium (80 g) and H_2_O (120 mL). The fungal strain was grown under static conditions at 20 °C for 28 days. The mycelium-containing culture medium was divided into small portions, extracted with AcOEt (3 × 5 L), and concentrated under reduced pressure to obtain the crude extract (53.2 g). The crude extract was separated into 7 fractions, Frs. 1–7, on a SiO_2_ column via step-gradient elution of CH_2_Cl_2_/MeOH (from 100:1 to 10:1, v/v). Fr. 4 (4.5 g) was separated by MPLC (MeOH–H_2_O from 20:80 to 100:0) into six subfractions, Frs. 4.1–4.6. Fr. 4.4 (119.3 mg) was further separated by CC (Sephadex LH-20; CH_2_Cl_2_–MeOH 1:1) followed by semi-prep HPLC (65% MeOH–H_2_O) to isolate compounds **1** (t_*R*_ 17.5 min; 9.5 mg) and **5** (t_*R*_ 22.5 min; 8.2 mg). Fr. 5 (6.5 g) was subjected to Sephadex LH-20 in CH_2_Cl_2_–MeOH (1:1) to isolate the major component, which was then separated by MPLC (MeOH–H_2_O from 10:90 to 100:0) to obtain Frs. 5.3.1–5.3.10. Fr. 5.3.6 (96.9 mg) was purified by semi-prep HPLC (75% MeOH–H_2_O) to yield compound **6** (t_*R*_ 18.0 min; 12.5 mg). Compounds **7** (t_*R*_ 31.5 min; 7.8 mg) and **8** (t_*R*_ 30.1 min; 11.3 mg) were isolated from Fr. 5.3.8 (67.0 mg) by semi-prep HPLC (50% MeCN–H_2_O). The separation of Fr. 6 (12 g) followed a procedure similar to that used for Fr. 5 to produce compounds **4**, **2**, and **3** by semi-prep HPLC (45% MeOH–H_2_O; t_*R*_ 27.0 min; 3.4 mg; 47% MeOH–H_2_O; t_*R*_ 19.0 min; 9.1 mg; and 50% MeOH–H_2_O; t_*R*_ 21.0 min; 7.6 mg, respectively).

### Chemical structure data

All investigated compounds were ≥95% pure (HPLC, wavelength = 210 nm). NMR data for compounds 3, 4 and 7 were completed for the first time in the present study. The NMR spectra of the compounds are provided in the Supporting Information.

Flavasperone (**1**): ^1^H-NMR (400 MHz, CDCl_3_): 2.48 (s, 3H), 3.90 (s, 3H), 3.95 (s, 3H), 6.26(s, 1H), 6.39 (d, *J* = 2.2 Hz, 1H), 6.57 (d, *J* = 2.2 Hz, 1H), 6.85 (s, 1H), 12.81 (s, 1H). HRESIMS: *m/z* [M+H] calcd for C_16_H_14_O_5_, 287.0875, found 287.0874.

Fonsecinone A (**2**): ^1^H-NMR (400 MHz, CDCl_3_): 2.10 (s, 3H), 2.46 (s, 3H), 3.41 (s, 3H), 3.59 (s, 3H), 3.76 (s, 3H), 4.01(s, 3H), 5.98 (s, 1H), 6.17(d, *J* = 2.2 Hz, 1H), 6.31 (s, 1H), 6.40 (d, *J* = 2.2 Hz, 1H), 6.95 (s, 1H), 7.03 (s, 1H), 12.81 (s, 1H), 15.23 (s, 1H). HRESIMS: *m/z* [M+H] calcd for C_32_H_26_O_10_, 570.1560, found 570.1562.

Fonsecinone C (**3**): ^1^H-NMR (400 MHz, CDCl_3_): 1.45 (s, 3H), 2.45 (s, 3H), 2.92 (dd, *J* = 17.0, 15.2 Hz, 2H), 3.38(s, 3H), 3.62 (s, 3H), 3.81 (s, 3H), 3.99 (s, 3H), 6.12 (d, *J* = 2.2 Hz, 1H), 6.31 (s, 1H), 6.36 (d, *J* = 2.2 Hz, 1H), 6.96 (s, 1H), 7.01 (s, 1H), 12.73 (s, 1H), 14.49 (s, 1H). ^13^C-NMR (100 MHz, CDCl_3_): 197.4 (C-4′), 183.1 (C-4), 166.9 (C-2), 165.3 (C-8′), 162.7 (C-8), 162.1 (C-6′), 160.3 (C-10), 156.8 (C-5), 155.8 (C-5′), 155.2 (C-10b), 151.4 (C-10′a), 142.8 (C-6a), 140.7 (C-9′a), 118.4 (C-9), 110.9 (C-5a), 109.7 (C-10a), 108.1 (C-5′a), 108.1 (C-3), 106.8 (C-10), 106.3 (C-4a), 103.7 (C-4′a), 102.2 (C-6), 100.4 (C-2′), 97.5 (C-7′), 96.7 (C-9′), 61.4 (10-OCH_3_), 56.4 (6′-OCH_3_), 56.2 (8-OCH_3_), 55.4 (8′-OCH_3_), 46.9 (C-3′), 29.3 (2′-CH_3_), 20.7 (2-CH_3_). HRESIMS: m/z [M+H] calcd for C_32_H_28_O_11_, 588.1665, found 588.1667.

Fonsecinone B (**4**): ^1^H-NMR (400 MHz, CDCl_3_): 1.78 (s, 3H), 2.09 (s, 3H), 2.99 (dd, *J* = 17.0, 15.2 Hz, 2H), 3.39(s, 3H), 3.60 (s, 3H), 3.73 (s, 3H), 3.99 (s, 3H), 5.94 (d, *J* = 8.1 Hz, 1H), 6.18(s, 1H), 6.38 (s, 1H), 6.68 (s, 1H), 6.82 (s, 1H), 14.13 (d, *J* = 2.2 Hz, 1H), 15.23 (d, *J* = 11.3 Hz, 1H). ^13^C-NMR (100 MHz, CDCl_3_): 196.7 (C-4′), 184.8 (C-4), 167.8 (C-2), 164.3 (C-8′), 162.9 (C-8), 161.6 (C-6′), 161.2 (C-6), 161.2 (C-5), 159.7 (C-5′), 153.5 (C-10a), 151.0 (C-10′a), 142.9 (C-9a), 140.9 (C-9′a), 117.0 (C-7), 110.9 (C-5a), 108.7 (C-5a′), 107.4 (C-3), 105.4 (C-10′),104.4 (C-4a), 103.7 (C-4′a), 102.9 (C-9), 102.1 (C-10), 100.3 (C-2′), 97.2 (C-7′), 96.6 (C-9′), 62.1 (6-OCH_3_), 56.4 (6′-OCH_3_), 56.1 (8-OCH_3_), 55.4 (8′-OCH_3_), 47.5 (C-3′), 29.0 (2′-CH_3_), 20.9 (2-CH_3_). HRESIMS: *m/z* [M+H] calcd for C_32_H_28_O_11_, 588.1665, found 588.1667.

Rubrofusarin B (**5**): ^1^H-NMR (400 MHz, CDCl_3_): 2.35 (s, 3H), 3.91 (s, 3H), 3.99 (s, 3H), 5.98 (s, 1H), 6.38 (d, *J* = 2.2 Hz, 1H), 6.56 (d, *J* = 2.2 Hz, 1H), 6.98 (s, 1H), 15.0 (s, 1H). HRESIMS: *m/z* [M+H] calcd for C_16_H_14_O_5_, 287.0875, found 287.0879.

Aurasperone A (**6**): ^1^H-NMR (400 MHz, CDCl_3_): 2.10 (s, 3H), 2.39 (s, 3H), 3.44 (s, 3H), 3.60 (s, 3H), 3.76 (s, 3H), 4.00(s, 3H), 5.96 (s, 1H), 6.03 (s, 1H), 6.18 (d, *J* = 2.2 Hz, 1H), 6.39 (d, *J* = 2.2 Hz, 1H), 6.95 (s, 1H), 7.13 (s, 1H), 14.82 (s, 1H), 15.23 (s, 1H). HRESIMS: *m/z* [M+H] calcd for C_32_H_26_O_10_, 570.1560, found 570.1557.

Aurasperone E (**7**): ^1^H-NMR (400 MHz, CDCl_3_): 1.47 (s, 3H), 2.38 (s, 3H), 2.92 (dd, *J* = 17.0, 15.2 Hz, 2H), 3.40(s, 3H), 3.61 (s, 3H), 3.79 (s, 3H), 3.97 (s, 3H), 6.01 (s, 1H), 6.09 (d, *J* = 2.2 Hz, 1H), 6.33 (d, *J* = 2.2 Hz, 1H), 6.94 (s, 1H), 7.09 (s, 1H), 14.52 (s, 1H), 14.77 (s, 1H). ^13^C-NMR (100 MHz, CDCl_3_): 197.8 (C-4′), 184.7 (C-4), 168.0 (C-2′), 165.1 (C-8), 162.3 (C-8′), 162.0 (C-6′), 161.9 (C-6), 160.4 (C-5), 153.3 (C-5′), 151.6 (C-10′a), 142.8 (C-10a), 140.4 (C-9a), 118.9 (C-9′a), 111.5 (C-7′), 110.4 (C-5′a), 107.9 (C-5a), 107.5 (C-3′), 106.6 (C-10), 104.9 (C-4′a), 104.0 (C-4a), 101.9 (C-9′), 101.5 (C-10′), 100.4 (C-2), 97.5 (C-7), 96.3 (C-9), 62.0 (6′-OCH_3_), 56.4 (6-OCH_3_), 56.1 (8′-OCH_3_), 55.4 (8-OCH_3_), 47.2 (C-3), 28.9 (2-CH_3_), 21.0 (2′-CH_3_). HRESIMS: *m/z* [M + H] calcd for C_32_H_28_O_11_, 588.1665, found 588.1667.

Asperpyrone C (**8**): ^1^H-NMR (400 MHz, CDCl_3_): 2.38 (s, 3H), 2.51 (s, 3H), 3.59 (s, 3H), 3.62 (s, 3H), 3.79 (s, 3H), 3.97 (s, 3H), 6.01 (s, 1H), 6.23(d, *J* = 2.2 Hz, 1H), 6.30 (s, 1H), 6.40 (d, *J* = 2.2 Hz, 1H), 6.95 (s, 1H), 7.09 (s, 1H), 13.09 (s, 1H), 14.72 (s, 1H); HRESIMS: *m/z* [M+H] calcd for C_32_H_26_O_10_, 570.1560, found 570.1558.

### Computational methods for ECD spectra

The conformational analyses were carried out for compounds **3**, **4** and **7** using BALLOON11 and confab12 programs. The BALLOON program searches conformational space with a generic algorithm, whereas the confab program systematically generates diverse low energy conformations that are supposed to be close to crystal structures. Conformations generated by both programs were grouped together by removing duplicated conformations in which the root mean square (RMS) distance was less than 0.5 Å. Semi-empirical PM3 quantum mechanical geometry optimisations were performed on the conformations using the Gaussian 0913 program. The duplicated conformations after geometry optimisation were then identified and removed. Remaining conformations were further optimised at the B3LYP/6-31G* level of theory in methanol solvent with the IEFPCM314 solvation model using the Gaussian 09 program, and duplicated conformations emerging after these calculations were removed according to the same RMS criteria indicated above. The harmonic vibrational frequencies were performed to confirm the stability of obtained conformers (Figures S106 and S107). Oscillator strengths and rotational strengths of the 20 weakest electronic excitations of each conformer were calculated using the TDDFT methodology at the B3LYP/6-311++G** level of theory with methanol as the solvent by the IEFPCM solvation model implemented in the Gaussian 09 program. The ECD spectra for each conformers were then simulated using Gaussian function with a bandwidth σ = 0.45 eV. The calculated spectra for each conformations were combined after Boltzmann weighting according to their population contributions.

### Antimicrobial activity

Whole-cell antimicrobial activity was determined by the broth microdilution^41^. Test strains were grown to the mid-log phase in Mueller–Hinton broth and diluted 1000-fold in the same medium. Cells (10^5^/mL) were inoculated into Mueller–Hinton broth and dispensed at 0.1 mL/well into a 96-well microtitre plate. MICs were determined in triplicate by serial 2-fold dilutions of test compounds. The MIC was defined as the concentration of a test compound that completely inhibited cell growth during a 24h incubation at 30 °C. Bacterial growth was determined by measuring the absorption at 600 nm using a microtitre ELISA reader.

### FabI cloning, expression, and purification

The full-length FabI gene (*E. coli* str. K-12 substr. MG1655, complete genome) was amplified from *E. coli* genomic DNA using the following forward and reverse primers: 5′-GGAATTCATATGGGTTTTCTTTCCGGTAAGCGC-3′ and 5′-GGGTGCCTCGAGTTATTTCAGTTCGAGTTCGTT-3′. The gene was cloned into a modified pET21b vector containing a 6 His-tag coding region at the N-terminus of the insert. After verifying the recombinant plasmids by sequencing, the plasmids were used to transform *E. coli* BL21 (DE3) cells. The transformed cells were grown in LB medium at 37 °C to an OD of 0.8–1.0 and induced with 0.5 mM isopropyl-D-thiogalactopyranoside (IPTG) at 18 °C for 18 h. The cells were harvested by centrifugation at 4,100 rpm for 10 min and re-suspended in lysis buffer containing 20 mM Tris, pH 8.5, 200 mM NaCl, and 10 mM imidazole, followed by disruption on a French press. Cell debris was removed by centrifugation at 21,000 rpm for 30 min. The protein was bound to Ni-agarose affinity resin, washed with buffer containing 20 mM Tris, pH 8.5, 200 mM NaCl, and 10 mM imidazole, and eluted with buffer containing 20 mM Tris, pH 8.8, 250 mM NaCl, and 150 mM imidazole. The protein was further purified by anion-exchange chromatography using a linear gradient of 10 mM to 1 M NaCl and size exclusion chromatography at pH 8.5 in 200 mM NaCl.

### Microscale thermophoresis

Recombinant *E. coli* FabI was labeled with the Monolith NT™ Protein Labeling Kit RED (Cat#L001) according to the supplied labeling protocol. Labelled FabI was kept constant at 50 nM, while all samples tested were diluted in a 20 mM HEPES (pH 7.4) and 0.05 (v/v)% Tween-20. After 10 min incubation at room temperature, samples were loaded into Monolith^TM^ standard-treated capillaries and the thermophoresis was measured at 25 °C after 30 min incubation on a Monolith NT.115 instrument (NanoTemper Technologies, München, Germany). Laser power was set to 20% or 40% using 30 seconds on-time. The LED power was set to 100%. The dissociation constrant Kd values was fitted by using the NTAnalysis software (NanoTemper Technologies, München, Germany).

### IC_50_ assay of compounds with *E. coli* FabI

NADH, NADPH, crotonoyl-CoA, and HEPES reagents were obtained from Sigma. All other chemicals were of analytical grade. The assay was performed in half 96-well microtiter plates. All compounds in this study were dissolved in DMSO to produce 100 mM stocks. The IC_50_ values of the investigated compounds were determined using 1 *μ*g of enzyme, 50 *μ*M crotonoyl-CoA as the substrate, and 100 *μ*M NADH as a cofactor^30^,^40^. Nine concentrations of the compounds were analyzed using a log-probit analysis program in GraphPad Prism 4.0. Equal volumes of DMSO and triclosan were used in the untreated and positive controls.

### Molecular Docking

For the small scale inverse docking^31^,^32^, crystal structures of docking targets (Table 2) were obtained from the Protein Data Bank (http://www.rcsb.org)^38^,^39^,^42^,^43^,^44^,^45^,^46^,^47^,^48^,^49^,^50^,^51^. The docking was performed by using ICM 3.8.2 modeling software on an Intel i7 4960 processor (MolSoft LLC, San Diego, CA)^52^. Ligand binding pocket residues were selected by using graphical tools in the ICM software, to create the boundaries of the docking search. In the docking calculation, potential energy maps of the receptor were calculated using default parameters. Compounds were imported into ICM and an index file was created. Conformational sampling was based on the Monte Carlo procedure^53^, and finally the lowest-energy and the most favorable orientation of the ligand were selected.

### Statistical analysis

Statistical analysis of the data was performed using Graph Pad Prism 4.0 software. The data were expressed as the means ± SD. Values were analyzed using SPSS version 12.0 software by one-way analysis of variance (ANOVA), and *p* < 0.05 was considered statistically significant.

## Additional Information

**How to cite this article**: He, Y. *et al*. Fungal naphtho-γ-pyrones: Potent antibiotics for drug-resistant microbial pathogens. *Sci. Rep.* **6**, 24291; doi: 10.1038/srep24291 (2016).

## Supplementary Material

Supplementary Information

## Figures and Tables

**Table 1:** *In vitro* antimicrobial activities of compounds **1–8.**

**Compounds**	**Minimum inhibitory concentrations (*****μ*****g/mL)**					
	**Gram-negative**				**Gram-positive**	
	***E. coli*****ATCC 35218**	***E. coli*****ATCC 25922**	***P. aeruginosa*****ATCC 27853**	***K. pneumoniae*****ATCC 700603**	***S. aureus*****ATCC 43300**	***E. faecalis*****ATCC 29212**
**1**	8.52	8.52	68.16	≥100	≥100	68.16
**2**	4.26	2.13	17.04	≥100	34.08	4.26
**3**	4.26	2.13	17.04	≥100	34.08	17.04
**4**	17.04	17.04	≥100	34.08	≥100	≥100
**5**	17.04	8.52	≥100	68.16	≥100	68.16
**6**	8.52	4.26	34.08	≥100	34.08	17.04
**7**	8.52	8.52	34.08	≥100	68.16	17.04
**8**	17.04	17.04	≥100	68.16	≥100	≥100
**Amikacin**	4.26	2.13	2.13	8.52	–	–
**Ceftriaxone**	8.52	2.13	2.13	2.13	–	–
**Vancomycin**	–	–	–	–	0.53	0.53

**Table 2:** Predicted binding free energies of compounds and target (ICM docking scores)^a^.

**PDB ID**^b^	**Protein name**	**Compounds**							
		**1**	**2**	**3**	**4**	**5**	**6**	**7**	**8**
2F9Y	AccA	−22.41	−9.62	−25.89	−16.63	−11.26	−22.15	−16.83	−9.48
1BDO	AccB	−16.23	−15.36	−16.93	−15.33	−14.74	−16.93	−25.01	−24.79
1BIA	AccC	−9.92	−15.96	−6.91	−17.24	−21.84	−19.17	−19.60	−20.82
1RQX	AccD	−12.68	−12.68	−13.32	−27.73	−3.11	−9.72	−14.40	−14.32
1NM2	FabD	−18.44	−14.46	−19.97	−28.08	−22.30	−14.04	−16.89	−15.84
4IIN	FabG	−16.95	−20.04	−18.22	−21.44	−9.40	−17.72	−25.69	−22.38
4KEH	FabA	−11.87	−18.10	−13.58	−12.46	−8.52	−12.20	−16.95	−10.80
4I83	FabZ	−8.10	−18.69	−13.01	−18.19	−13.52	−7.03	−22.96	−13.71
1LXC	FabI	−19.95	−37.40	−35.27	−20.57	−19.20	−31.41	−30.62	−16.38
1MFP	FabK	−17.60	−17.51	−15.88	−25.96	−21.29	−16.87	−7.071	−21.63
3OJF	FabL	−21.87	−18.84	−22.88	−18.97	−18.09	−14.66	−25.09	−13.96
2VB9	FabB	−18.24	−14.66	−20.25	−18.44	−20.02	−15.19	−10.83	−27.47
2GFW	FabF	−6.51	−21.57	−15.45	−23.67	−15.95	−11.18	−14.70	−14.05
1TXT	FabH	−17.82	−16.31	−15.71	−10.33	−10.78	−24.36	−16.01	−16.37

^a^Docking score/interaction potential of compounds with targets (kcal/mol).

^b^See refs 38,39,42, 43, 44, 45, 46, 47, 48, 49, 50, 51.

**Table 3:** Binding affinity and inhibitory activities of screened compounds **1–8.**

**Compound**	**Dissociation constant with** ***E. coli*** **FabI**	**Inhibitory activities against** ***E. coli*** **FabI**
	**Kd**^a^ (***μ*****M)**	**IC**_**50**_(***μ*****M)**
1	bn.i.	110.1
2	215.0 ± 28.8	2.7
3	270.0 ± 9.1	3.0
4	bn.i.	110.7
5	bn.i.	cn.i.
6	289.0 ± 31.1	52.1
7	329.0 ± 29.7	71.8
8	bn.i.	cn.i.
Triclosan	11.4 ± 8.3	0.77

^a^The Kd value is automatic calculated by the curve fitting, and presents as means ± SD for three experiments.

^b^n.i. is no clear binding detected in the MST measurement.

^c^n.i. is no inhibition detected in the experiments (IC_50_ > 1 mM).
