# Supplementary material for: Fungal naphtho-γ-pyrones: Potent antibiotics for drug-resistant microbial pathogens
Source: Sci Rep. 2016 Apr 11;6:24291. doi: 10.1038/srep24291 (PMC4827027; doi:10.1038/srep24291)
Supplement: Supplementary Information [file srep24291-s1.pdf]

Supporting Information

Fungal naphtho-γ-pyrones: Potent antibiotics for drug-resistant microbial pathogens

Yan He[1], Jun Tian[2], Xintao Chen[1],Weiguang Sun[1], Hucheng Zhu[1], Qin Li[1], Liang Lei[1], Guangmin Yao[1], Yongbo Xue[1], Jianping Wang[1]*, Hua Li[1]*, Yonghui Zhang[1]*

[1] Hubei Key Laboratory of Natural Medicinal Chemistry and Resource Evaluation, School of Pharmacy, Tongji Medical College, Huazhong University of Science and Technology, Wuhan 430030, China.

[2] Department of Physical Medicine and Rehabilitation, Zhongnan Hospital of Wuhan University, Wuhan 430071, China.

**Corresponding author.*

* Tel./fax:+86-027-83692892

Emails: zhangyh@mails.tjmu.edu.cn (Y. Z.)

li_hua@mail.hust.edu.cn (H. L)

jpwang1001@163.com (J.W.)

Table of Contents

**Complete experiment flow diagram**	3
Figure S1	3
**Spectrum of the compounds 1-8**	4-13
Figure S2**:** ^{1}H NMR spectrum ($CDCl_3$, 400 MHz) of Flavasperone	4
Figure S3: ^{13}C NMR spectrum ($CDCl_3$, 100 MHz) of Flavasperone	4
Figure S4: EI-MS spectrum of Flavasperone	4
Figure S5: ^{1}H NMR spectrum ($CDCl_3$, 400 MHz) of Fonsecinone A	5
Figure S6: ^{13}C NMR spectrum ($CDCl_3$, 100 MHz) of Fonsecinone A	5
Figure S7: ^{1}H NMR spectrum ($CDCl_3$, 400 MHz) of Fonsecinone C	6
Figure S8: ^{13}C NMR spectrum ($CDCl_3$, 100 MHz) of Fonsecinone C	6
Figure S9: ^{1}H NMR spectrum ($CDCl_3$, 400 MHz) of Fonsecinone B	7
Figure S10: ^{13}C NMR spectrum ($CDCl_3$, 100 MHz) of Fonsecinone B	7
Figure S11: ^{1}H NMR spectrum ($CDCl_3$, 400 MHz) of Rubrofusarin B	8
Figure S12: ^{13}C NMR spectrum ($CDCl_3$, 100 MHz) of Rubrofusarin B	8
Figure S13: EI-MS spectrum of Rubrofusarin B	8
Figure S14: ^{1}H NMR spectrum ($CDCl_3$, 400 MHz) of Aurasperone A	9
Figure S15: ^{13}C NMR spectrum ($CDCl_3$, 100 MHz) of Aurasperone A	9
Figure S16: ^{1}H NMR spectrum ($CDCl_3$, 400 MHz) of Aurasperone E	10
Figure S17: ^{13}C NMR spectrum ($CDCl_3$, 100 MHz) of Aurasperone E	10
Figure S18: ^{1}H NMR spectrum ($CDCl_3$, 400 MHz) of Asperpyrone C	11
Figure S19: ^{13}C NMR spectrum ($CDCl_3$, 100 MHz) of Asperpyrone C	11
Figure S20: Experimental ECD spectra of compounds **2**–**4** and **6**–**8**	12
Figure S21: Experimental and calculated ECD spectra of compound **3**	12
Figure S22: Experimental and calculated ECD spectra of compound **4**	12
Figure S23: Experimental and calculated ECD spectra of compound **7**	13
**Others**	
Figure S24: Pictures of *Aspergillus* sp. Z120 on PDA medium.	13
ITS sequence of *Aspergillus* sp. Z120.	13
Figure S25: Enzymes involved in the fatty acids metabolism	14
Figure S26: Low-energy binding conformations and predicted binding free energies of cephalochromin bound to *E.coli* FabI generated by virtual ligand docking.	14
Table S1: Predicted binding free energies of compounds **1**–**8** and four unrelated target.	14

Complete experiment flow diagram

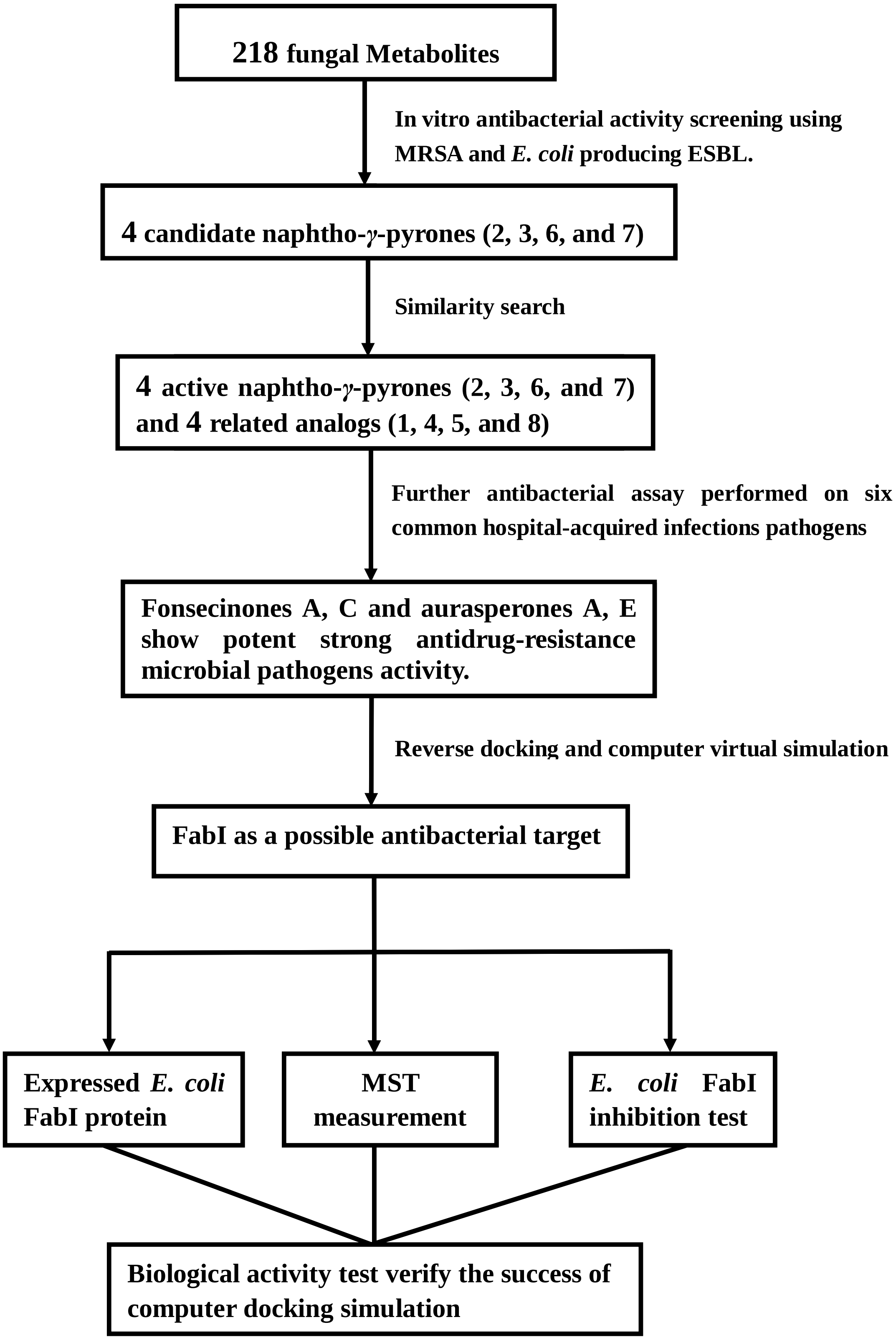

Figure S1. In vitro antibacterial screening，computer docking simulation and bioassay evaluation flow diagram.

Spectrum of the test compounds

Flavasperone (Compound 1)

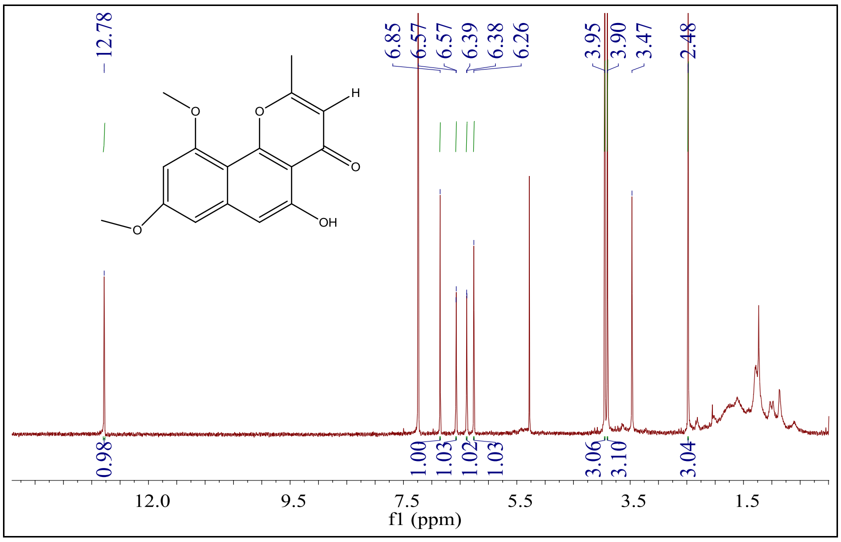

Figure S2: ^{1}H NMR spectrum ($CDCl_3$, 400 MHz) of Flavasperone

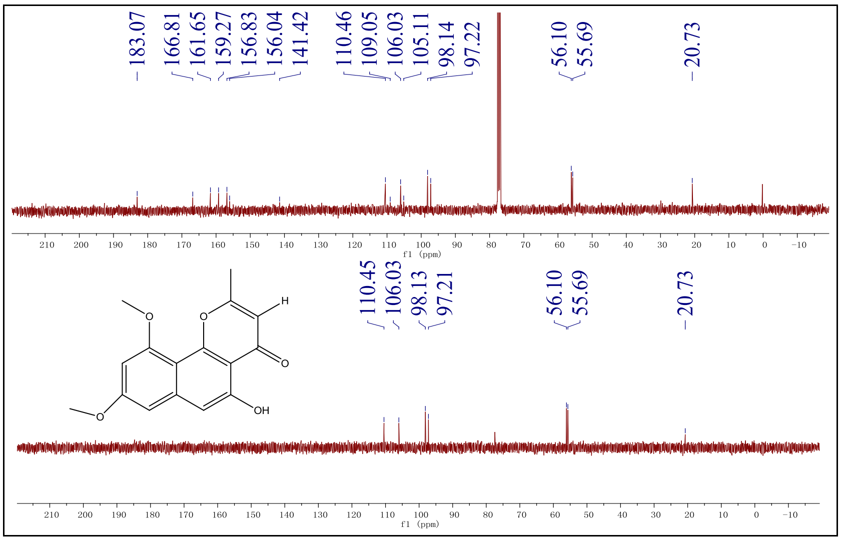

Figure S3: ^{13}C NMR spectrum ($CDCl_3$, 100 MHz) of Flavasperone

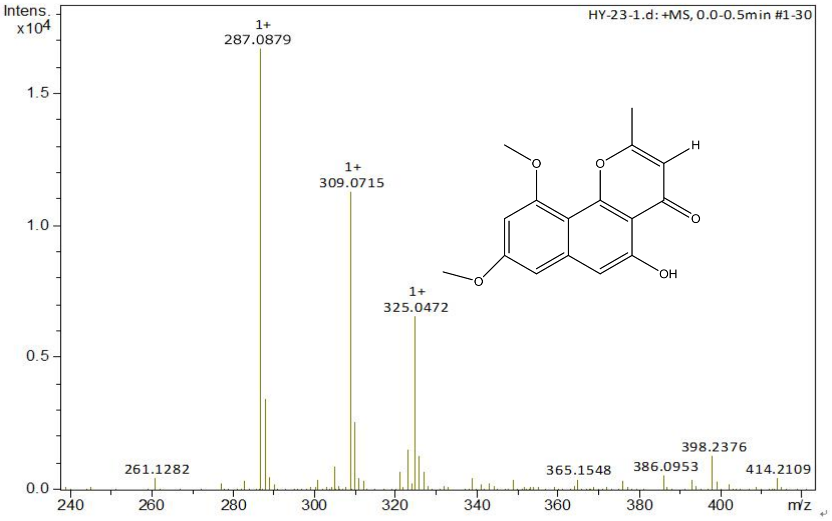

Figure S4: EI-MS spectrum of Flavasperone

Fonsecinone A (Compound 2)

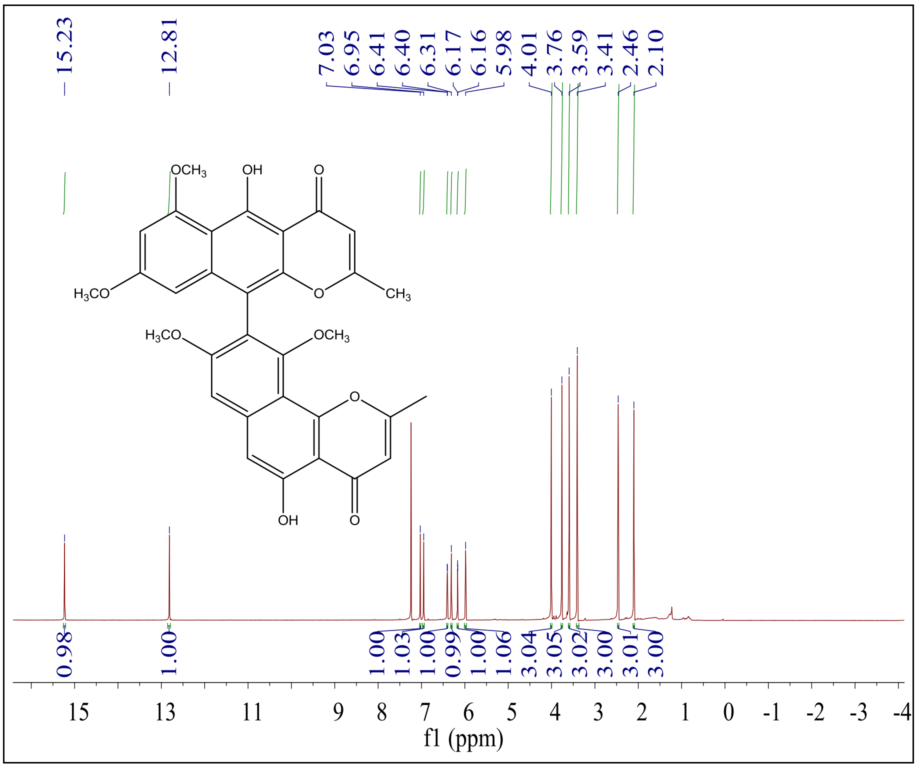

Figure S5: ^{1}H NMR spectrum ($CDCl_3$, 400 MHz) of Fonsecinone A

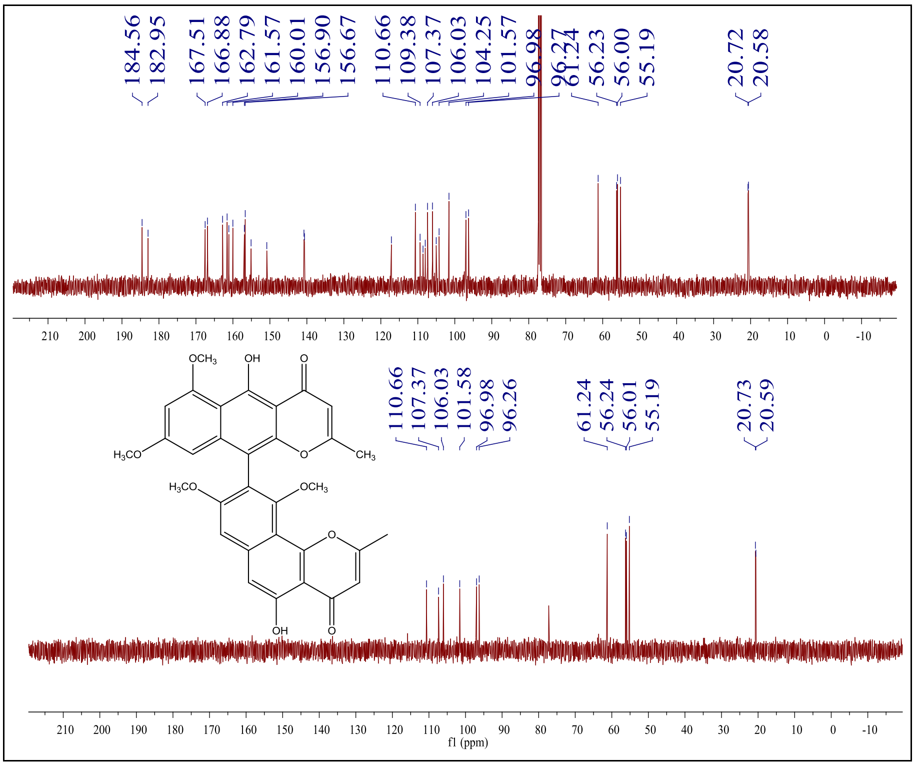

Figure S6: ^{13}C NMR spectrum ($CDCl_3$, 100 MHz) of Fonsecinone A

Fonsecinone C (Compound 3)

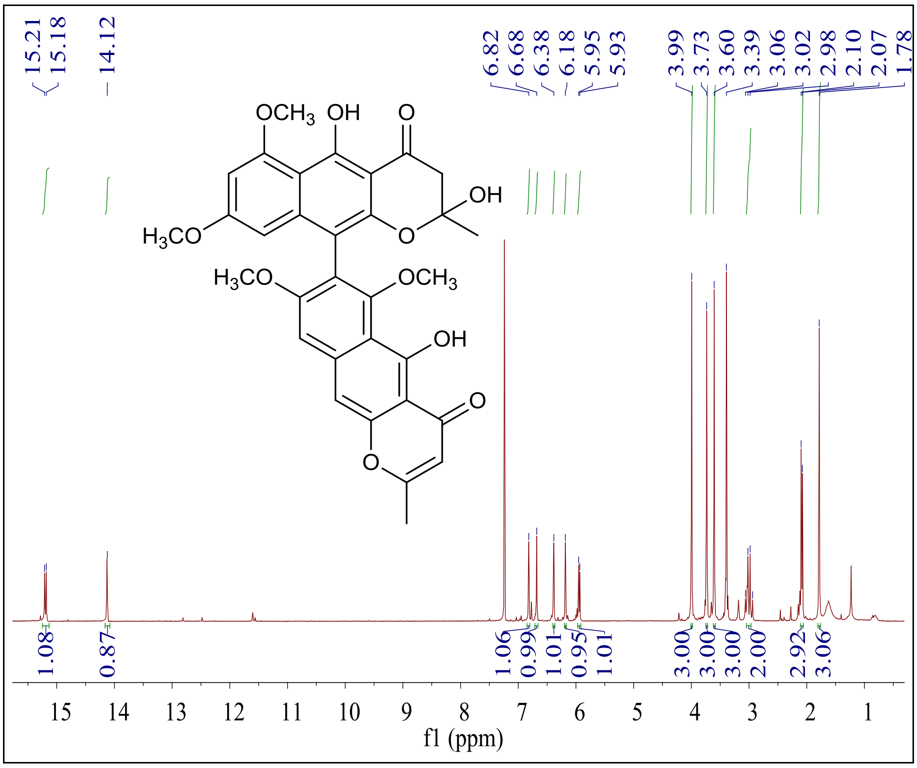

Figure S7: ^{1}H NMR spectrum ($CDCl_3$, 400 MHz) of Fonsecinone C

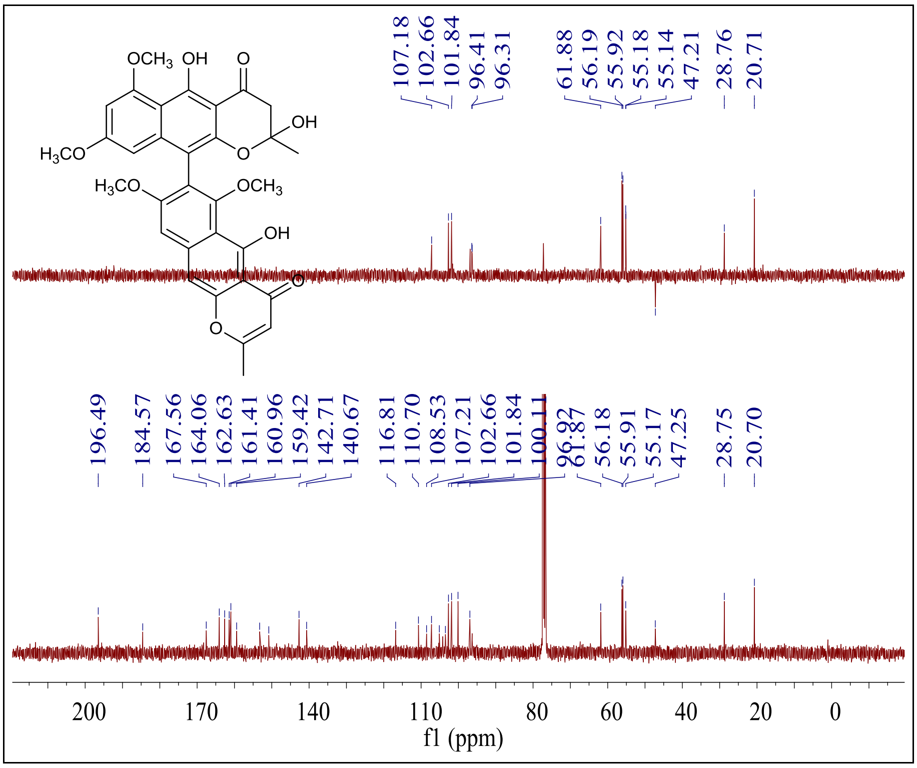

Figure S8: ^{13}C NMR spectrum ($CDCl_3$, 100 MHz) of Fonsecinone C

Fonsecinone B (Compound 4)

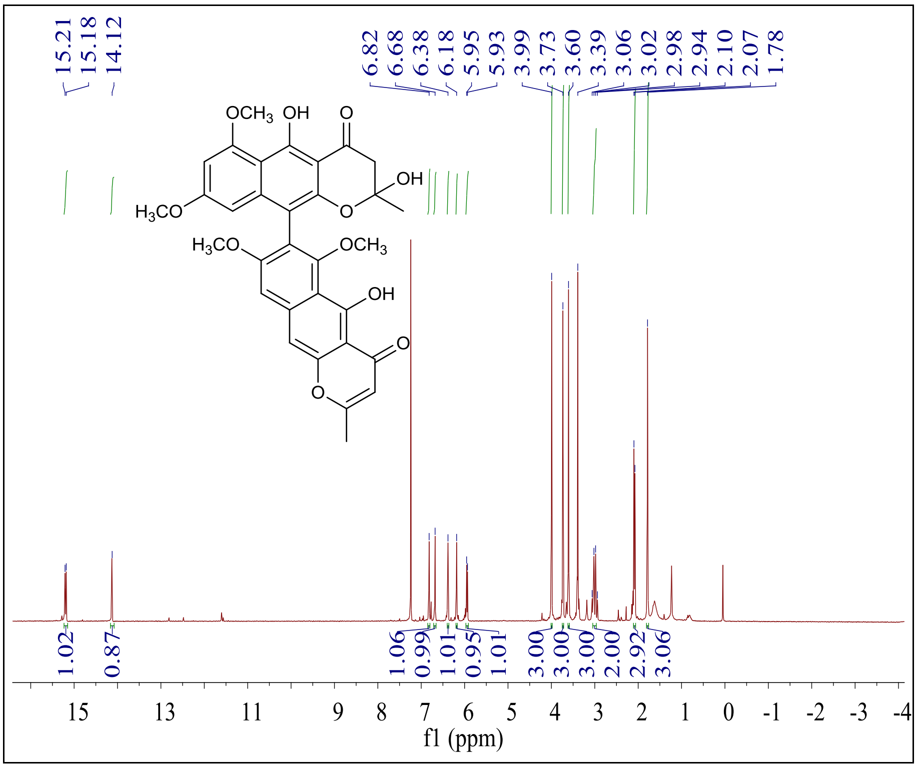

Figure S9: ^{1}H NMR spectrum ($CDCl_3$, 400 MHz) of Fonsecinone B

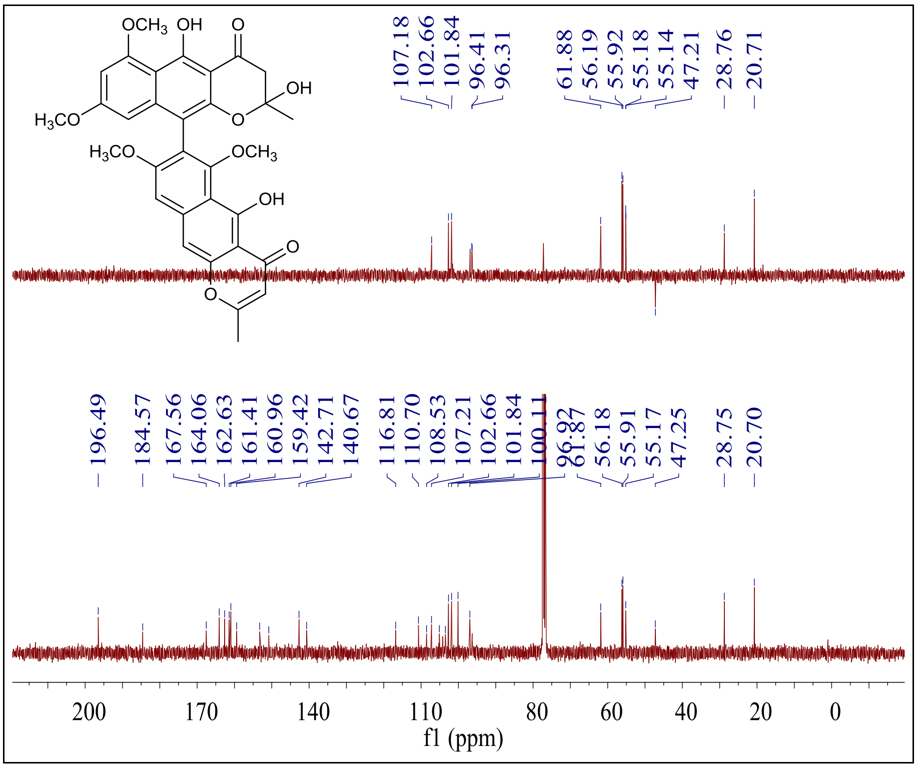

Figure S10: ^{13}C NMR spectrum ($CDCl_3$, 100 MHz) of Fonsecinone B

Rubrofusarin B (Compound 5)

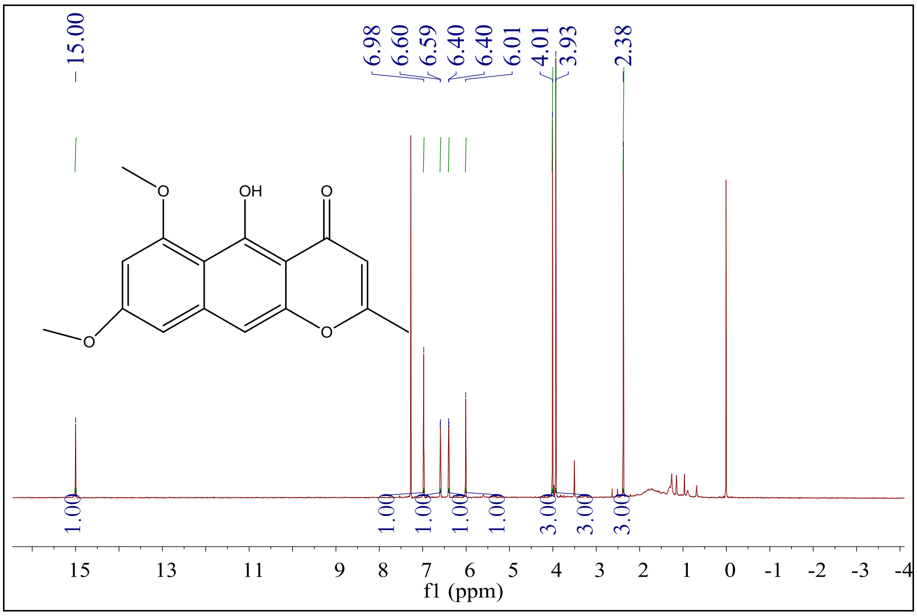

Figure S11: ^{1}H NMR spectrum ($CDCl_3$, 400 MHz) of Rubrofusarin B

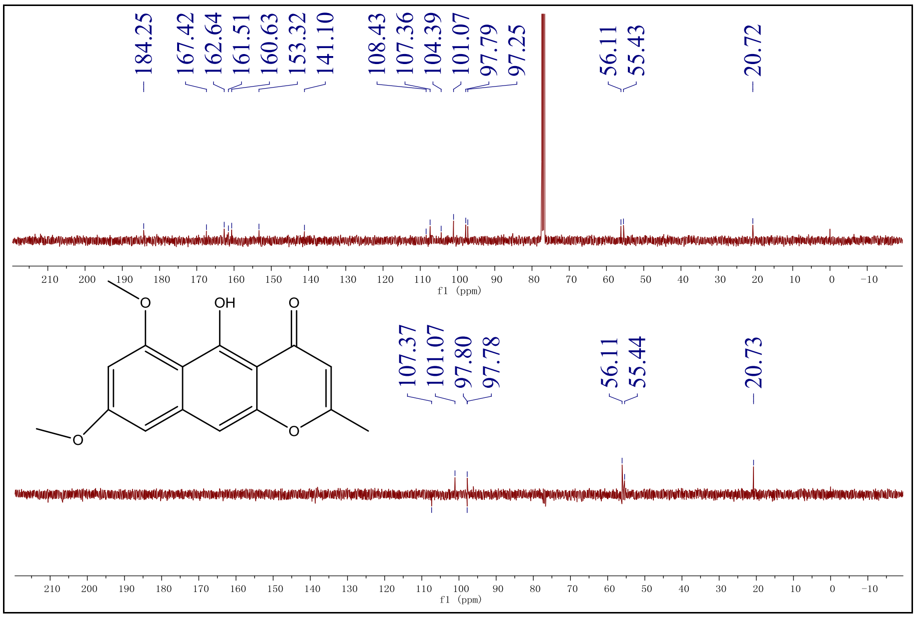

Figure S12: ^{13}C NMR spectrum ($CDCl_3$, 100 MHz) of Rubrofusarin B

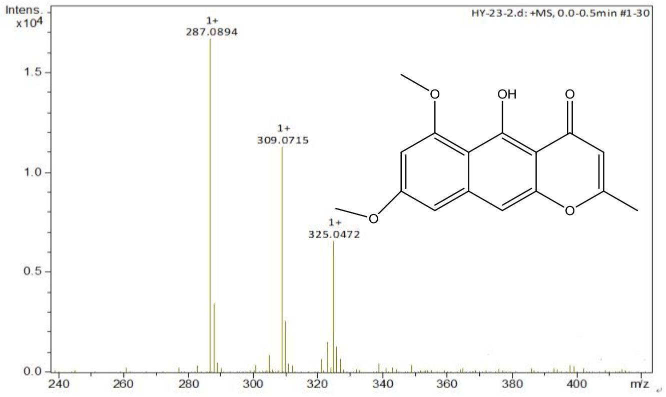

Figure S13: EI-MS spectrum of Rubrofusarin B

Aurasperone A (Compound 6)

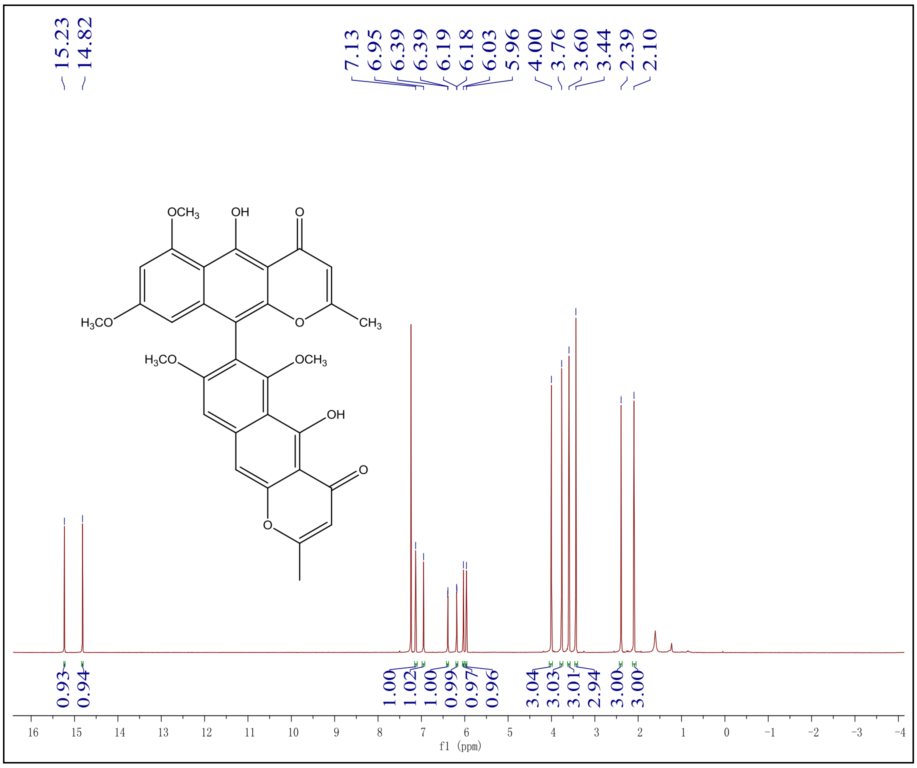

Figure S14: ^{1}H NMR spectrum ($CDCl_3$, 400 MHz) of Aurasperone A

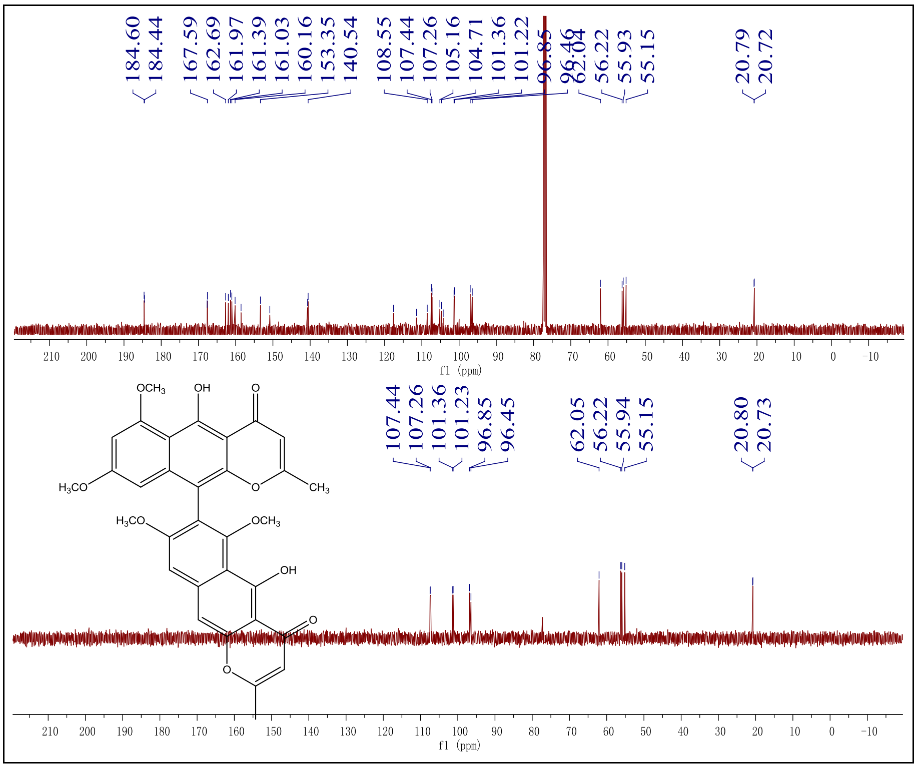

Figure S15: ^{13}C NMR spectrum ($CDCl_3$, 100 MHz) of Aurasperone A

Aurasperone E (Compound 7)

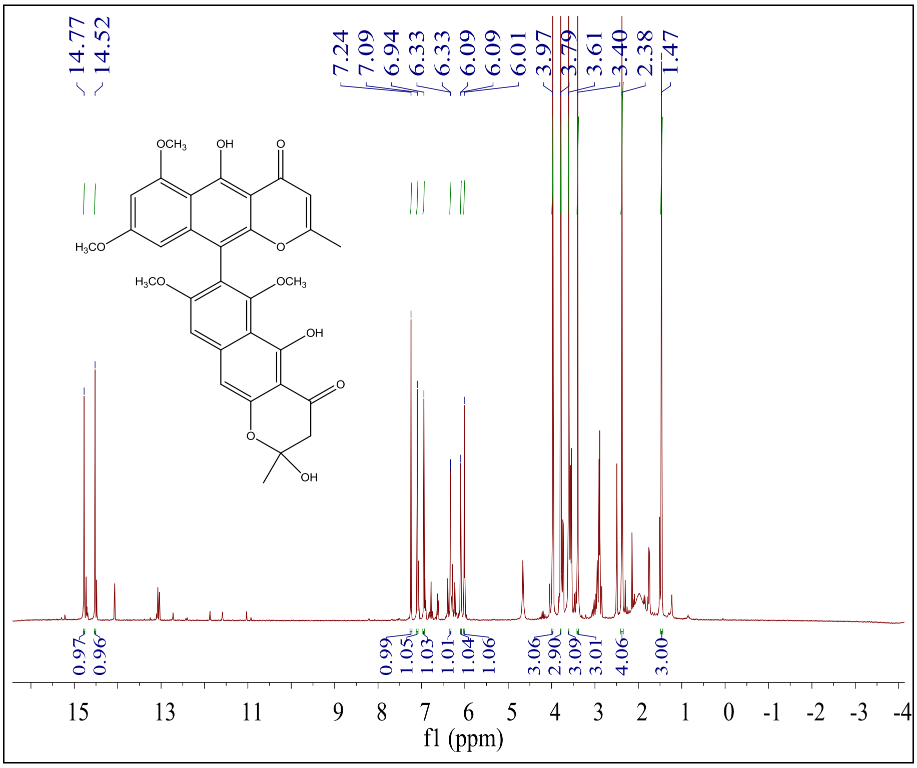

Figure S16: ^{1}H NMR spectrum ($CDCl_3$, 400 MHz) of Aurasperone E

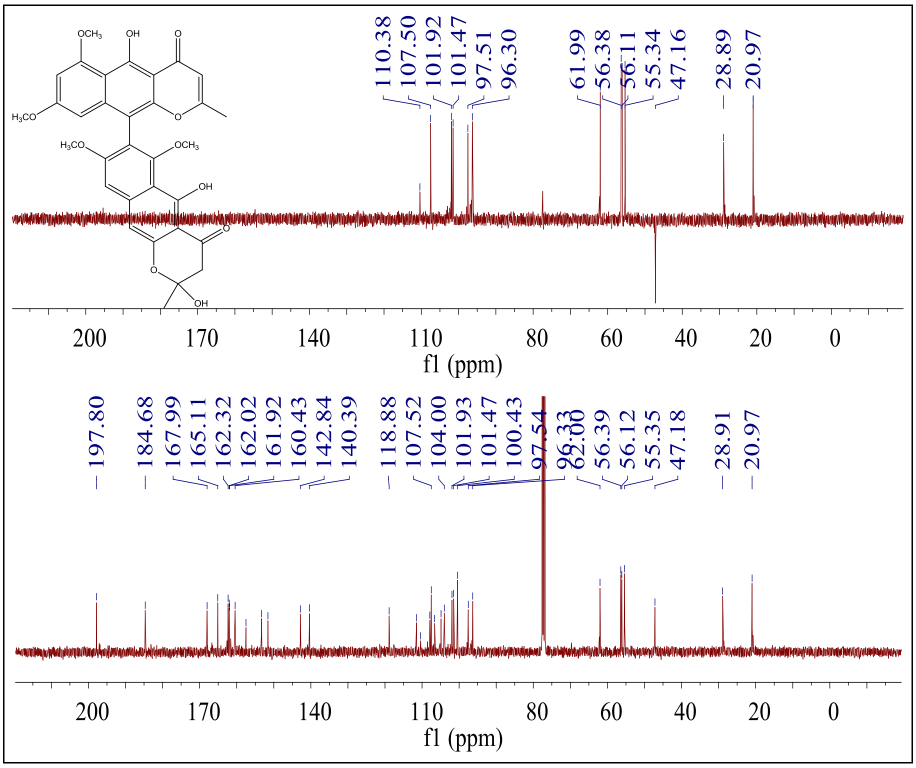

Figure S17: ^{13}C NMR spectrum ($CDCl_3$, 100 MHz) of Aurasperone E

Asperpyrone C (Compound 8)

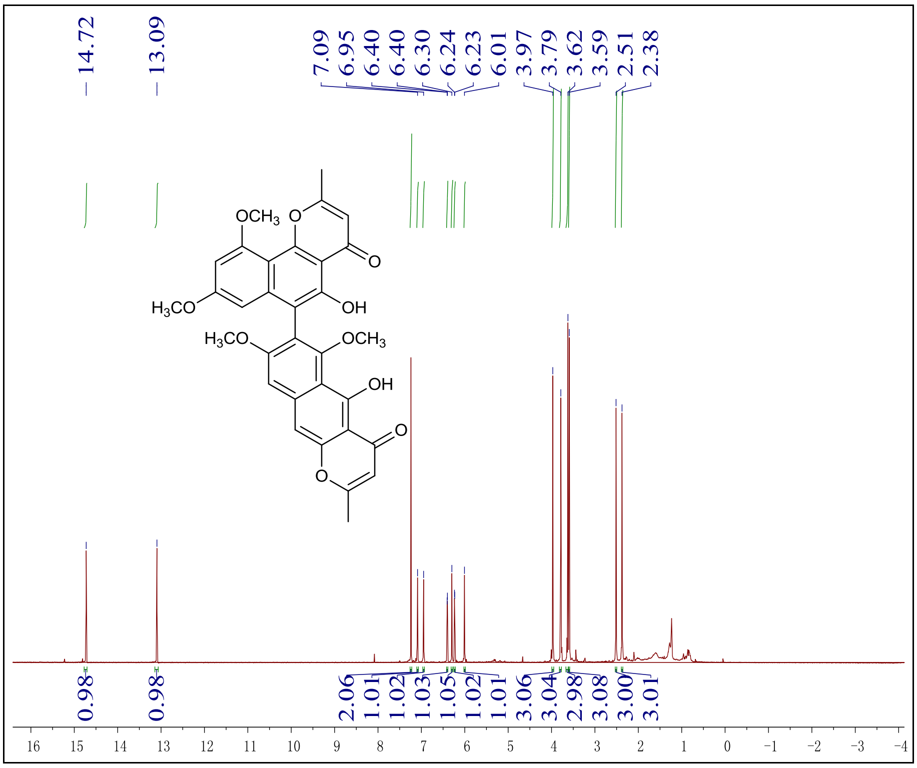

Figure S18: ^{1}H NMR spectrum ($CDCl_3$, 400 MHz) of Asperpyrone C

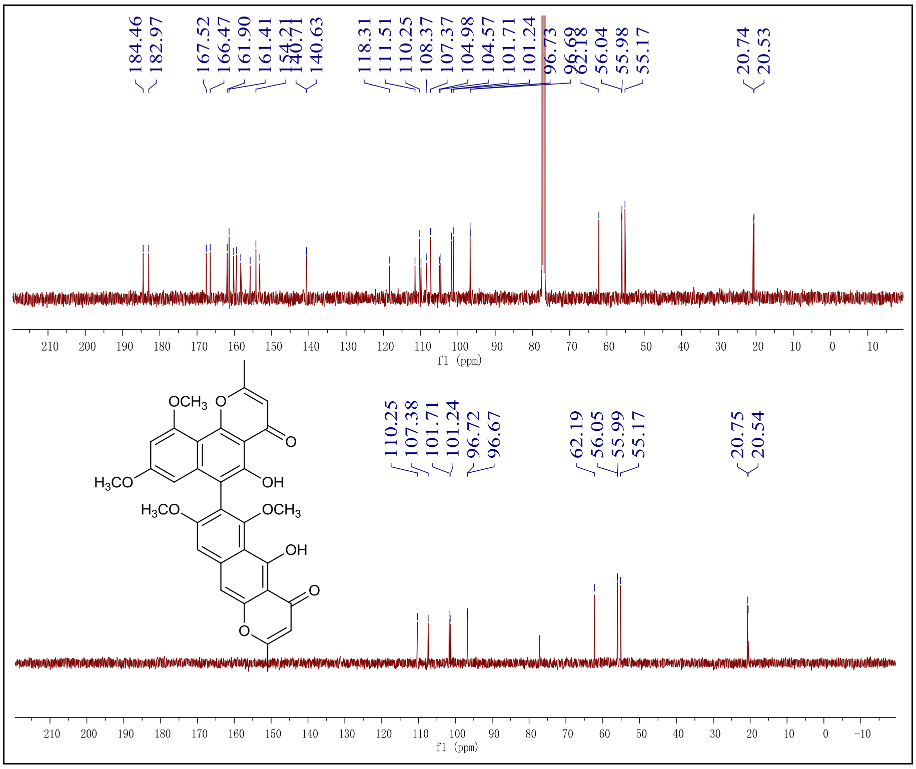

Figure S19: ^{13}C NMR spectrum ($CDCl_3$, 100 MHz) of Asperpyrone C

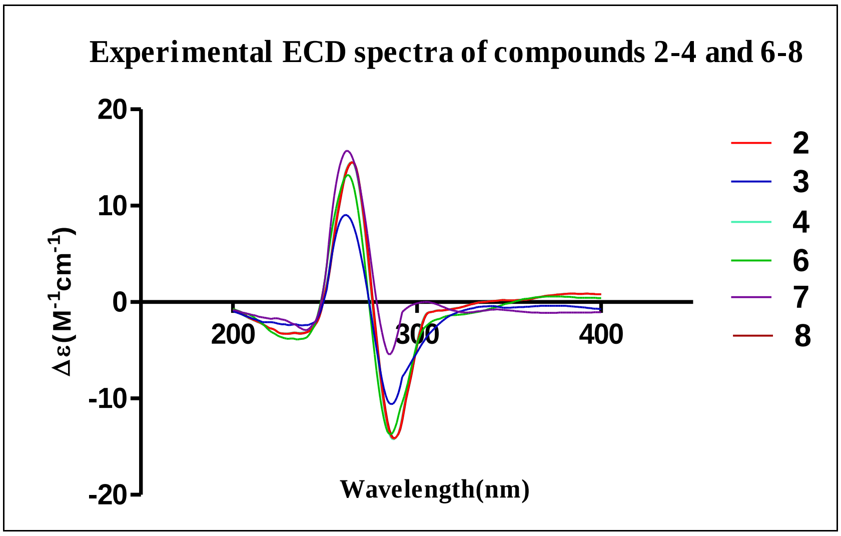

Figure S20: Experimental ECD spectra of compounds **2**–**4** and **6**–**8**

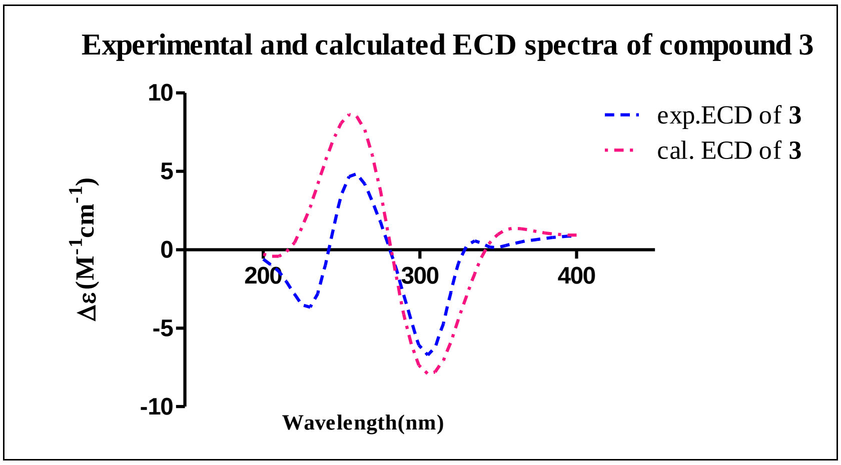

Figure S21: Experimental and calculated ECD spectra of compound **3**

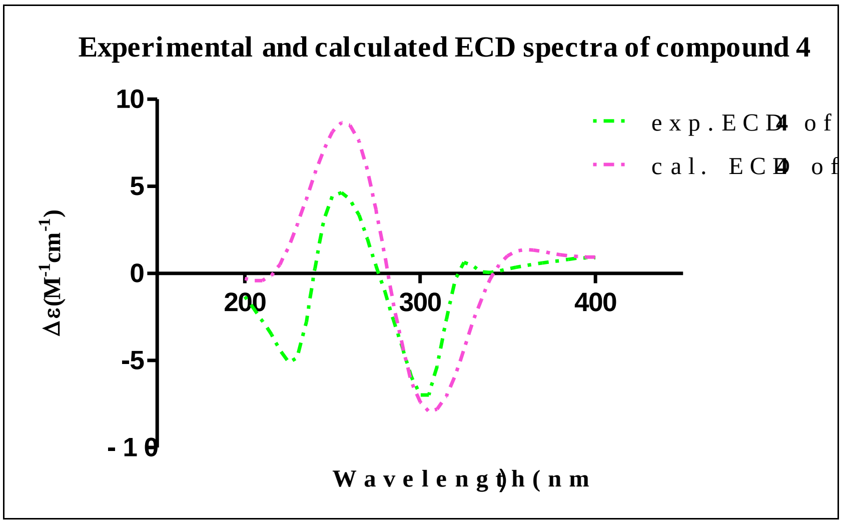

Figure S22: Experimental and calculated ECD spectra of compound **4**

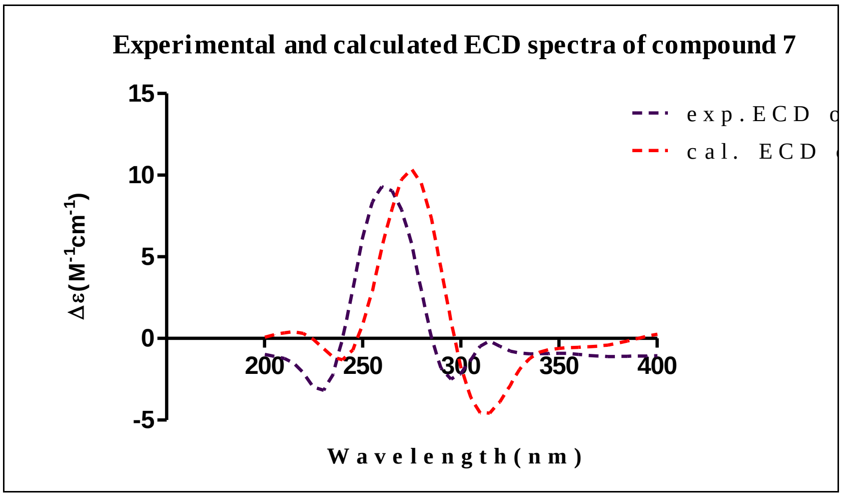

Figure S23: Experimental and calculated ECD spectra of compound **7**

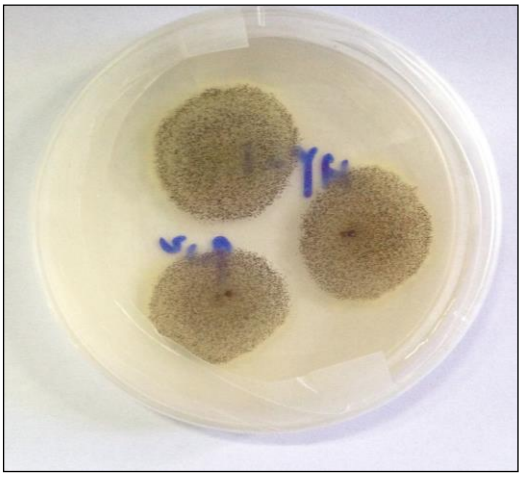

Figure S24: Pictures of the colony of *Aspergillus* sp. Z120 on PDA medium.

ITS sequence of *Aspergillus sp*. Z120

TTTGGGCcCAACCTCCCATCCGTGTCTATTGTACCCTGTTGCTTCGGCGGGCCCGCCGC
TTGTCGGCCGCCGGGGGGGCGCCTCTGCCCCCCGGGCCCGTGCCCGCCGGAGACCCC
AACACGAACACTGTCTGAAAGCGTGCAGTCTGAGTTGATTGAATGCAATCAGTTAAAA
CTTTCAACAATGGATCTCTTGGTTCCGGCATCGATGAAGAACGCAGCGAAATGCGATA
ACTAATGTGAATTGCAGAATTCAGTGAATCATCGAGTCTTTGAACGCACATTGCGCCCC
CTGGTATTCCGGGGGGCATGCCTGTCCGAGCGTCATTGCTGCCCTCAAGCCCGGCTTGT
GTGTTGGGTCGCCGTCCCCCTCTCCGGGGGGACGGGCCCGAAAGGCAGCGGCGGCAC
CGCGTCCGATCCTCGAGCGTATGGGGCTTTGTCACATGCTCTGTAGGATTGGCCGGCGC
CTGCCGACGTTTTCCAACCATTCTTTCCAGGTTGACCTCGGATCAGGTAGGGATACCCG
CTGAACTTAAGCATATcAATAAGCGGAGGA

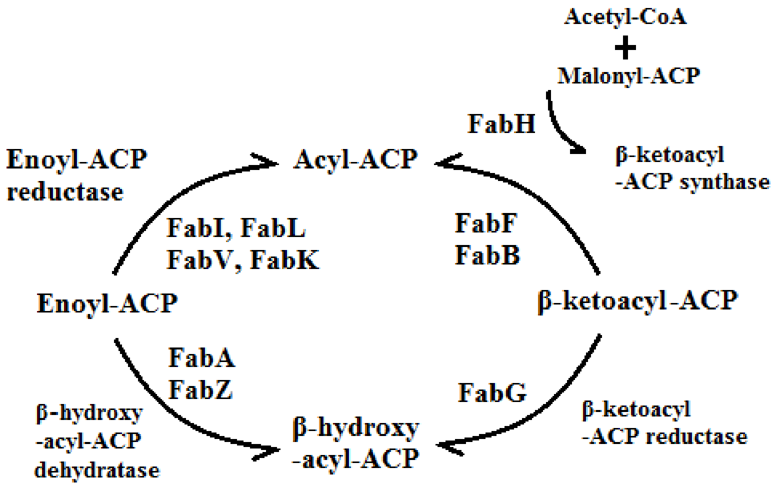

Figure S25: Enzymes involved in the fatty acids metabolism

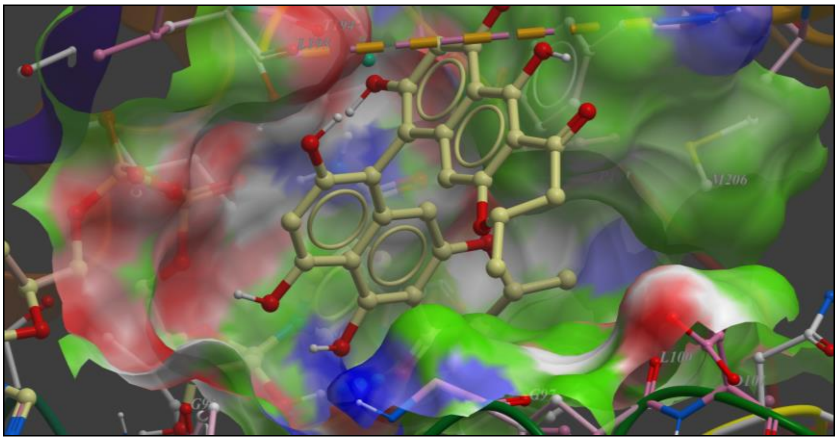

Figure S26: Low-energy binding conformations of cephalochromin bound to *E.coli* FabI generated by virtual ligand docking. The structure of FabI was depicted in ribbon form. Cephalochromin are depicted as the ball-and-stick model showing carbon (yellow), hydrogen (grey), oxygen (red) atoms. The ICM docking scores is —29.23 kcal/mol.

Table S1 Predicted binding free energies of compounds **1—8** and four unrelated target (ICM docking scores)[a]

PDB ID[6-9]	Protein name	Compounds							
		1	2	3	4	5	6	7	8
2ZC3	PBP-2X	−9.31	−5.78	−19.07	−12.25	−19.1	−19.83	−12.67	−16.83
3VSK	PBP-3	−7.10	−12.68	−24.83	−21.54	−23.42	−21.1	−25.33	−10.06
4FDO	DprE1	−7.86	−12.19	−18.53	−23.59	−11.88	−19.74	−4.24	−16.89
4UMX	IDH1_R132H	−6.72	−5.29	−11.24	−25.21	−9.65	−5.22	−14.12	−13.06

[a] Docking score/interaction potential of compounds with targets (kcal/mol).
